# Cardiac magnetic resonance circumferential strain predicts myocardial fibrosis in DMD-associated cardiac disease

**DOI:** 10.1186/1532-429X-15-S1-O35

**Published:** 2013-01-30

**Authors:** Kan N Hor, Elizabeth Perkins, Michael Taylor, Linda Cripe, Kathleen Lao, Wojciech Mazur, Subha V Raman, D Woodrow Benson

**Affiliations:** 1 Pediatric Cardiology, Cincinnati Children's Hospital Medical Center, Cincinnati, OH, USA; 2 Pediatrics, Nationwide Children's Hospital, Columbus, OH, USA; 3 Pediatrics, Children's Hospital of Wisconsin, Milwaukee, WI, USA; 4 Internal Medicine/Cardiology, Ohio State University, Columbus, OH, USA; 5 Internal Medicine/Cardiology, The Christ Hospital, Cincinnati, OH, USA

## Background

Duchenne Muscular Dystrophy (DMD) is an X-linked recessive disease with an incidence of 1/3500 live male births. Loss of the dystrophin protein leads to progressive muscle degeneration and an absolute genetic risk of developing cardiac disease. Currently there is a lack of a sensitive diagnostic tool to identify cardiac dysfunction in early stages of the disease and standard measures of decreased global function, e.g. ejection fraction (EF), are only detectable when advanced disease is present. We found that myocardial circumferential strain (Ecc, an indicator of local myocardial deformation normalized to its original dimension) can detect occult cardiac disease early in the course of disease despite normal EF, and Ecc magnitude further declines with advancing age and disease progression. DMD associated cardiac disease leads to replacement of normal myocyte with myocardial fibrosis leading to eventual cardiac dysfunction; cardiac magnetic resonance (CMR) has been used to assess for late gadolinium enhancement (LGE, an indicator of myocardial fibrosis) in DMD patients. We sought to determine if Ecc is a predictor of myocardial fibrosis in DMD patients.

## Methods

We evaluated 2 serial CMR studies from genetically confirmed DMD patients. In Group A, the first and second studies were LGE negative, and in Group B patient the first study was LGE negative and the second study was LGE positive. Standard imaging analysis for EF was performed using (QMASS (Medis Medical Imaging Systems, Leiden, the Netherlands) and tagged imaging was performed using HARP (Diagnosoft, Palo Alto, California) software. DMD patients were categorized into two groups. Statistical analysis were performed using Student's t-test to compare Ecc between the two groups.

## Results

We evaluated 39 patients: Group A (n=16, 32 studies) and Group B (n=23, 46 studies). There was no difference in age (14.4 ± 2.8 vs 13.8 ± 2.9 years) or EF (63.6 ± versus 60.2 ± 7.9 percent) between the two groups. At the first study both groups were LGE negative, but there was a statistically significant difference in Ecc magnitude between Group A and Group B (-15.6 ± 1.7 vs. 14.2 ± 2.1, p = 0.002). Serial changes in age or EF were not different between the two groups, and the Ecc in Group A patients did not decrease significantly over the study period (-16.2 ± 1.5 versus -15.1 ± 1.8). However, the Ecc magnitude in Group B patients decreased over the same study period (-14.8 ± 1.9 versus -13.6 ± 2.1, = 0.04).

## Conclusions

These studies illustrate the heterogeneity of progression of DMD-associated cardiac disease. These results suggest that Ecc alone may not predict the presence of LGE. However, the combination of Ecc and LGE prediction development of EF abnormality and provide a sensitive primary outcome of a therapeutic trial.

## Funding

None.

